# Production of hydrogen from crude glycerol via an integrated process of L-malate biosynthesis by *Escherichia coli* and photofermentation by *Rhodobacter capsulatus*

**DOI:** 10.1186/s12934-025-02866-y

**Published:** 2025-11-10

**Authors:** Jose L. Marcos, Gema Cabrera, Daniel F. Hernandez, Beatriz Luque, Antonio Valle, Jorge Bolivar

**Affiliations:** 1 Department of Biomedicine, Biotechnology and Public Health-Biochemistry and Molecular Biology, Campus Universitario de Puerto Real, University of Cadiz, Cadiz, Spain; 2 Department of Chemical Engineering and Food Technology, University of Cadiz, Campus Universitario de Puerto Real, Cadiz, Spain; 3 International Campus of Excellence (ceiA3), Institute of Viticulture and Agri-Food Research (IVAGRO), University of Cadiz, Cadiz, Spain; 4 Institute of Biomolecules (INBIO), University of Cadiz, Cadiz, Spain

**Keywords:** Biorefinery, Biodiesel, Crude glycerol, L-malate, Bio-H_2_, Escherichia coli, Rhodobacter capsulatus.

## Abstract

Biorefinery is an innovative concept for resource utilization. To further increase its efficiency, the integration of different biomass conversion technologies (integrated biorefinery) is essential. Biodiesel, a traditional biofuel, generates glycerol as an inevitable byproduct; making it an attractive carbon source for biorefineries due to its abundance, low price, and high degree of reduction. In this work, we propose an integrated two-step process of dark fermentation with *Escherichia coli* to produce L-malate from glycerol and photofermentation by *Rhodobacter capsulatus* to convert this L-malate into hydrogen, a clean and sustainable energy carrier. To this end, we optimized an *E. coli* L-malate-producing strain by overexpressing GlpK in the M4-Δ*iclr*/pBAD-*pck* strain and scale up the process from shake flask to bioreactor using waste crude glycerol from a biodiesel biorefinery instead of pure glycerol. Under the optimized conditions, *E. coli* produced up to 11 g/L L-malate in 24 h. The culture medium from this dark fermentation was used to formulate an L-malate enriched medium for *Rhodobacter* that led to the production of 58.0 ± 6 mM H_2_ in 90 h.

**Graphical abstract:**

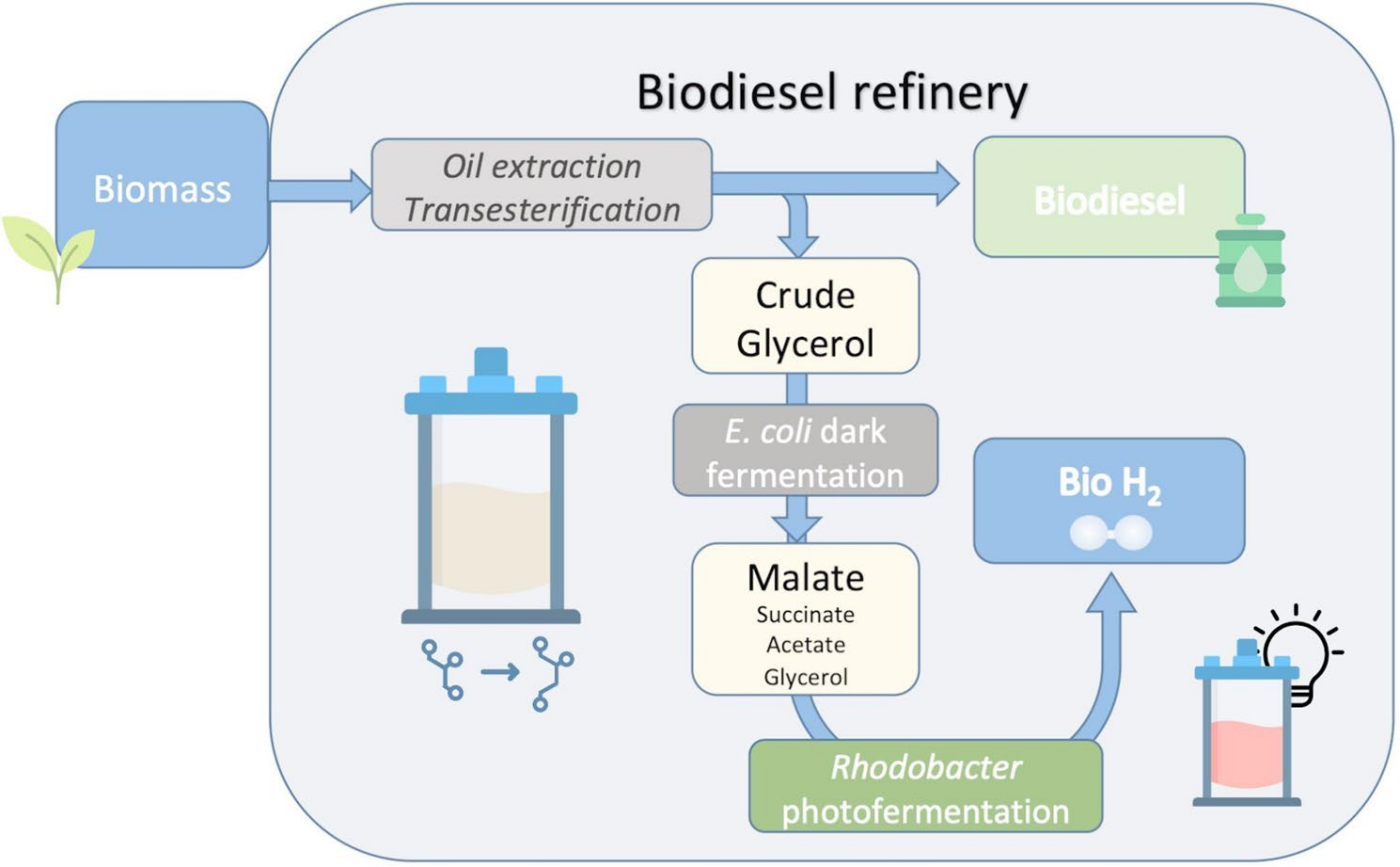

**Supplementary Information:**

The online version contains supplementary material available at 10.1186/s12934-025-02866-y.

## Introduction

The use of renewable resources in industry applying biorefinery processes is essential to decrease environmental damage. Biorefineries are processing facilities that convert biomass into value-added products such as biofuels, biochemicals, bioenergy, and other biomaterials [1]. The biorefinery concept is a pioneering approach that fully supports the principles of sustainability and environmental responsibility, and it should be seen as a paradigm in which social, economic, environmental, and quality-of-life considerations are balanced. To further enhance their efficiency, the integration of various biomass conversion technologies (integrated biorefinery) is essential. Process integration at the design stage is key to further improving economic and environmental performance [2].

Biomass is the only carbon-neutral renewable resource for the sustainable production of traditional liquid biofuels such as biodiesel, bio-ethanol, and bio-butanol. Biomass feedstocks, animal fats, plants, or recycled oils containing fatty acids have been used to generate biofuels, although during this process, numerous by-products are generated [3]. With a 70% increase in production between 2005 and 2015, biodiesel has a much larger global market than other biofuels. Hydrotreated vegetable oil (HVO), also known as green diesel, is one of the most widely used biodiesels and significant increases in its production are projected by 2030 [4]. However, in biodiesel industry, glycerol is generated as an unavoidable by-product, accounting for 10% of total biodiesel [5].

This has made glycerol an attractive carbon source for biorefineries due to its abundance, low price, and high degree of reduction. Therefore, the conversion of crude glycerol into value-added products is essential to increase the economic viability of the biofuel industry in a context of a circular economy [6]. In this sense, the biotransformation of glycerol into other high-value chemicals and biofuels has been proposed [3, 5, 7–12]. Among them, the 1,4-dicarboxylic (C4) acid L-malate is a commercially important chemical with a wide range of applications in cosmetics, pharmaceuticals, textile finishing, metal cleaning, or as a food additive in beverages, among others [13, 14]. Pure glycerol has shown good results as a carbon source for biotransformation to L-malate by some microorganisms, although with insufficient yields to scale up the process. Consequently, metabolic engineering and/or adaptive evolution have been used to optimize novel microbial strains with improved L-malate yield and productivity or to obtain new L-malate-producing microorganisms [15, 16].

For example, the biotransformation of pure glycerol by the fungus *Ustilago trichophora* was reported to increase L-malate yield to 0.67 g malate/g glycerol after adaptive evolution and metabolic engineering [17, 18]. In another case, metabolic engineering and a fed-batch strategy with the yeast *Yarrowia lipolytica*, improved the fermentation of pure glycerol from 56 g/L L-malate in Flasks up to 112 g/L in 5 L bioreactor at 144 h, although in this case a complex medium containing yeast extract and peptone was used [19]. However, few works using only crude glycerol −the actual by-product of biodiesel biorefineries− for L-malate production have been carried out. Biotransformation of glycerol by the fungal *Aspergillus niger* is one of the few examples where crude glycerol has already been tested, proving that it could be used as an effective substrate for fungal L-malate production [20]. Culture optimization and batch fermentation led to 0.25 yield after 192 h of fermentation. A methodology for L-malate recovery from the culture was later described [14, 21]. L-malate-producing fungal strains offer advantages such as secretion efficiency, natural aerobic fermentation, and pH tolerance [22, 23]. However, filamentous growth, long fermentation times, and complex growth requirements hinder the production process [24].

On the other hand, bacterial fermentation, specifically in *Escherichia coli*, overcomes fungal production disadvantages since growth is usually faster and cheaper, and bioreactor handling is easier to scale [25, 26]. *E. coli* can metabolize glycerol anaerobically, producing a mixture of organic acids (succinate, formate, acetate, and lactate) and ethanol to maintain redox balance. Nevertheless, the wild-type strains are unable to produce and export L-malate to the culture medium [27]. Several metabolic engineering strategies have been carried out to force *E. coli* to produce L-malate both in anaerobiosis [28] and in aerobiosis [29, 30]. These strategies usually involve the depletion or inactivation of competitive pathways for the synthesis of fermentative end products, activation of the glyoxylate shunt, deletion of tricarboxylic acid (TCA) cycle genes, and overexpression of anaplerotic enzymes [16, 27, 30]. Most of this work has been carried out using glucose as a carbon source. Nevertheless, we have previously engineered the M4-*ΔiclR/pck E. coli* strain to produce L-malate (5.25 g/L) and succinate (1.79 g/L) from 12.5 g/L pure glycerol in 48 h [27]. Considering acetate production (1.05 g/L), conversion from consumed glycerol to exported organic acids (C4 and acetate) represented a 65% yield (g/g). In this strain, the synthesis of acetate was restricted by deleting the acetate kinase phosphotransacetylase (*ackA-pta*) and pyruvate oxidase (*pox*) genes. The TCA cycle was also split into two linear pathways by deletion of the succinate dehydrogenase (*sdhA*) gene, which encodes one of the catalytic subunits (the FAD-binding protein), impairing the synthesis of fumarate. In this mutant background, L-malate production in the reductive branch was enhanced by the activation of the glyoxylate shunt (through the deletion of the repressor, *iclR* gene) and the overexpression of the enzyme Pck [27].

Although L-malate is considered a versatile chemical building block, its downstream purification presents challenges due to economic and environmental issues. Several processes have been reported for this purpose, including liquid-liquid extraction with organic solvents [13] and anion-exchange chromatography [31]. However, these processes are expensive and hinder industrial production. To avoid or reduce the need for downstream processing, the L-malate-enriched culture media from bacterial fermentation can be used as a carbon source for other microorganisms of industrial interest. For example, it can be used to produce bio-hydrogen (bio-H_2_), which is another form of bioenergy recognized as the most promising replacement for fossil fuels [12, 32, 33]. Bio-H_2_ is produced by several types of bacteria in anaerobic conditions. These bacteria include chemoheterotrophs (dark fermentation, DF) and photoheterotrophs (photofermentation, PF), such as purple non-sulfur bacteria (PNSB). The use of these bacteria to produce bio-H_2_ is considered an emerging technology [33, 34]. Although PNSB have a great degree of flexibility for adaptation to a broad variety of substrates, they clearly display an affinity towards the short chain fatty acids (SCFAs) or volatile fatty acids (VFAs), namely, acetate, butyrate, formate, lactate, L-malate, propionate, succinate, pyruvate, etc. Photoheterotrophic bacteria, can utilize these acids as electron donors for H_2_ production depending on light energy. The rate of growth of PNSBs, substrate conversion efficiency, and H_2_ production rate significantly change with the type of substrate. It is well documented that PNSBs produce hydrogen at better rates from organic acids than pure sugars [35]. For example, the PNSB *Rhodobacter capsulatus* uses organic compounds as electron donors in the presence of light to produce H_2_ in a nitrogen-limiting condition and an optimum C/N ratio to dissipate the excess energy and reducing power [36, 37]. However, the bottleneck of this process is the expensive costs of the pure organic acids (acetate, lactic acid, succinate, L-malate, etc.) that this microorganism requires to produce H_2_. A strategy to solve this problem is the combination of DF and PF in a two-step fermentation process. For example, DF can be used in a biodiesel refinery to transform crude glycerol into volatile fatty acids (VFAs), which are the preferred substrates for producing bio-H_2_ using PNSBs [38]. Many photo-fermentative biohydrogen studies with *Rhodobacter* species have used L-malate or succinate as the organic substrate under an optimum carbon-to-nitrogen ratio in batch reactors [37].

This work aims to develop a two-step fermentation process for producing H₂, involving the synthesis of L-malate from crude glycerol via DF using *E. coli*, followed by the PF of this L-malate using *R. capsulatus*. To this end, metabolic engineering was first used to improve the DF process in the M4-*ΔiclR/pck E. coli* strain. Then, the operating conditions in bioreactors were optimized for crude glycerol using a state-of-the-art microbioreactor platform. Finally, the L-malate-enriched medium from DF was used to formulate an L-malate culture medium for the production of bio-H_2_ by *R. capsulatus*.

## Experimental procedures

### Plasmids, strains, and chemicals

The open reading frame of the *E. coli glpK* gene was cloned into the inducible expression vectors pBAD-18-Cm (pB-Cm) using standard methods [39] . This gene was amplified from BW25113 genomic DNA as template using the Q5 High-Fidelity polymerase (New England Biolabs Inc.) and then cloned using the appropriate restriction enzymes. DNeasy^®^ Microbial^®^ Pro (Qiagen) DNA extraction, Nucleospin^®^ Clean-up, and Nucelospin^®^ Plasmid kits (Macharey-Nagel) were used for DNA isolation and purification. The *E. coli* Δ*sdhA*Δ*ack-pta*Δ*pox*∆*iclR* mutant strain, hereafter referred to as M4-∆*iclR* [27], was used as the parental strain for transformation of the vector abovementioned as well as the pBAD-Kan-*pck* (pB-K*-pck*) [27]. The knocked-out genes and overexpression vectors were verified by PCR using GoTaq^®^ polymerase (PROMEGA). The P_BAD_ promoter was induced with L-arabinose at 0.02% (w/v) (= 0.013 mM) [40]. For the photofermentation assays, the *Rhodobacter capsulatus* S2 mutant strain with hydrogenase deletions (S2 Δ*hupAB*, P_hupA_:::lacZ (Rif ^R^ Kan ^R^), was used [41]. The *E. coli* and *R. capsulatus* strains, plasmids, and oligonucleotides are listed in the Supporting Information Table S1. Antibiotics were purchased from Sigma-Aldrich (Merck KGaA, Darmstadt, Germany); L-arabinose, IPTG, mineral salts, and other chemicals were purchased from VWR^®^, Sigma^®^, and Thermo^®^ Scientific. Crude glycerol was obtained from the biodiesel factory Abengoa Bioenergía (S.A.), San Roque (Cádiz) [42, 43].

### L-malate production assays using pure glycerol in Erlenmeyer flasks

*Escherichia coli* strains were initially streaked from − 80 °C glycerol stocks on Luria-Bertani (LB) agar plates. The pre-inoculum was prepared by transferring one colony to 5 ml LB broth, to which antibiotics chloramphenicol (Cm) and/or kanamycin (Kan) were added when appropriate, at final concentrations of 25 and 50 µg/ml, respectively, and incubating overnight. Then, cultures were centrifuged at 6000 ×* g* and resuspended in 50 mL M9 medium with antibiotics in 250 mL Erlenmeyer Flasks. M9 culture media composition for 1 L was: 9.97 g Na_2_HPO_4_, 3 g KH_2_PO_4_, 1 g NH_4_Cl, 0.5 g NaCl, 4 g of NaHCO_3_, 2 mL MgSO_4_ 1 M, 0.1 mL CaCl_2_ 1 M, 1 mL of thiamine (1 mg/mL) and 0.1 mL trace element solution (1.15 g/L FeCl_3_-6H_2_O, 0.0195 g/L CuSO_4_-5H_2_O, 0.0245 g/L ZnSO_4_-7.H_2_O, 0.16 g/L MnCl_2_-7.H_2_O, 0.0645 g/L CoCl_2_- 6H_2_O, 0.0185 g/L (NH_3_) 6 Mo_7_O_24_-4.H_2_O and 0.125 g/L H_3_BO_3_). Pure glycerol was added to a final concentration of ∼12,5 g/L in all the pre-inoculum cultures.

After overnight culture, biomass was centrifuged at 6000 ×*g* and transferred to fresh 50 mL M9 without antibiotics at an initial OD_570_ ∼0.1, and was induced with L-arabinose and/or IPTG at OD_570_ ∼0.6. All the assays were incubated at 37 °C in a rotary shaker at 200 rpm, and samples were withdrawn at 0, 6, 24, 48, and 72 h as previously described in [27].

### Experimental conditions of the full-factorial design for L-malate production using* E. coli*

A full-factorial screening design of experiment (DoE) was performed to identify independent factors that significantly influenced the L-malate molar yield (mol L-malate per mol glycerol consumed) at 24 h as response variable using the engineered M4-∆*iclR*/*pck*-*glpK* strain. Two levels were evaluated for each factor, and 3 central points for a total of 11 experiments, including three replicates of the central point (level 0) for the three factors studied in this work. These replicates are needed to analyze the variability of the experimental procedure and to validate the statistical study of the experimental results. The factors evaluated were inoculum biomass (OD_570_ = 0.10, 0.60, and 1.10), dissolved oxygen (DO) controlled at 20%, 60%, and 100% air saturation, and glycerol concentration in the initial culture medium (9, 12, and 15 g/L). Experimental assays were carried out in 3 mL using the 24-well microbioreactors system micro-Matrix (Applikon^®^ Biotechnology, Delft, Netherlands) at the Institute of Biomolecules (INBIO) facilities (Universidad de Cádiz). A colony of *E. coli* strain was picked out in 20 mL of LB with antibiotics, and the overnight culture was centrifuged, and the pellet was resuspended in 150 mL of M9 medium with appropriate antibiotics, and 12 g/L of pure or crude glycerol. This culture was induced at OD_570_ ∼0.15 and grown overnight. The next day, the culture was centrifuged, and the biomass was resuspended in M9 with pure or crude glycerol at an initial OD_570_ of 0.1, 0.6, or 1.1. Data acquisition of temperature (^o^C), DO, and pH were monitored every minute during 24 h. The experimental design and statistical analysis were carried out using Statgraphics Centurion v. 19. Detailed information on the DoE and results can be found in the Supporting Information, Table S2 and Fig. S1.

### L-malate production assays in 0.5 L scale mini-bioreactors

The experimental procedure for inoculation was carried out as described in Sect. "Experimental conditions of the full-factorial design for L-malate production using E. coli".The initial OD_570_ in the bioreactor culture was ∼1.1 (= 0.363 g CDW/L). M9 culture was supplemented with pure or crude glycerol as described in the results and discussion section. The DF experiments were performed in the 0.5 L Minibio Bioreactor System (Applikon^®^ Biotechnology B.V., Delf, Netherlands) at the INBIO facilities with a working volume of 150 mL. This bioreactor enables real-time monitoring and control of pH, dissolved oxygen, and temperature. Lucullus^®^ Software was used for control of variables and data acquisition. After each fermentation, the culture medium was removed from the bioreactor, centrifuged at 20,000 x g, and the obtained supernatant was filtered through 0.22 nm nylon filter for storing at -20 °C for further analysis and application in photofermentation.

### Metabolic model refinement and in silico approach for flux balance analysis (FBA) of the engineered E. coli strains using pure or crude glycerol

A base *E. coli* core model for the K-12 MG1655 strain was downloaded from BIGG [44] and used for in silico analysis. To construct the in silico model for the M4 mutant, reactions were added to the base model in order to incorporate glycerol metabolism: glycerol transport (GLYCt), glycerol kinase (GLYK) and G3PD2 (glycerol 3-P dehydrogenase). In addition, reactions for glucose assimilation (EX_glc_D_e) were removed because glucose was absent in the medium. The acetate kinase (ACKr), phosphate transacetylase (PTAr), and succinate dehydrogenase (SUCDi) reactions, were also removed to reproduce the genetic knock-outs of the M4-iclR mutant. The specific rates for (i) glycerol uptake, (ii) succinate, L-malate, and acetate production, and (iii) biomass growth were calculated using the raw data obtained from Flasks and Bioreactor assays and inserted into the model as flux rates. All flux rates were normalized to a glycerol flux of -20 mmol / g CDW h. To close the carbon flux, all excess or missing carbon was compensated by taking into account CO_2_ uptake/production. The averages of all the trial data at the 24-hour time point were set as constraints in the models. Metabolic fluxes were then calculated using parsimonious FBA (pFBA). The methods were implemented in Python using the COBRApy library [45]. Escher-FBA was used to visualize and perform interactive flux balance analysis in *E. coli* metabolic map [46].

### Photofermentation of L-malate from DF with rhodobacter capsulatus

*Rhodobacter capsulatus* was firstly cultivated in photoheterotrophic/chemoheterotrophic conditions by picking up a colony from a Petri dish into 25–30 mL of PY medium containing: 10 g/L tryptone or peptone, 0.5 g/L yeast extract, 0.75 g/L MgSO_4_·7 H_2_O, 0.15 g/L CaCl_2_ anhydrous, 1 mg/L thiamine, and 1 mL trace elements prepared previously in solution stock: 400 mg MnSO_4_ · H_2_O, 700 mg H_3_BO_3_, 10 mg Cu(NO_3_)_2_ · 3 H_2_O, 60 mg ZnSO_4_ · 7 H_2_O, 190 mg Na_2_MoO_4_ · 7 H_2_O and 250 mL distilled H_2_O [41]. Rifampicin (50 µg/mL) and kanamycin were also added. The cultures were grown up to 70 h. The grown biomass was centrifuged at 4 °C, 6,000 x g for 10 min. The pellet was resuspended and used to inoculate the RCV L-malate-medium containing 1, 2, 3, 4, 5, 6–8 g/L of L-malate from DF and the following mineral salts in 1 L: 1.0 g KH_2_PO4, 0.5 g K_2_HPO_4_, 0.2 g MgSO_4_·7 H_2_O, 0.022 g of anhydrous CaCl_2_, 0.01 g FeSO_4_·7 H_2_O, 1 mL trace element solution previously mentioned and 1 mg/L thiamine [47]. All media were adjusted to pH 6.8 with 5 M NaOH. For the bio-H_2_ fermentation, 8.8 ml of inoculated medium was added to 22 ml vials at an initial OD_570_ of 0.3 and sealed with a rubber septum for hermetic closure. The inoculation was performed in a glove box that was sparged with nitrogen to maintain an oxygen concentration of 2–4%. All the assays were incubated at 30 °C without shaking. The LED light intensity was set at 150 µmol photons / m^2^·s^1^ (33 W/m^2^) by measuring with a digital luxmeter (SQ-420X Smart Quantum Sensor). Unit photon flux was converted into “W/m^2^” according to the factor (0.20–0.23 W/m^2^ = 1 µmol photons m^2^·s^1^). This conversion gives the radiant energy within the PAR range (400–700 nm) for a full-spectrum LED grow light [48].

### Analytical procedures

L-malate, succinate, acetate, and glycerol were quantified from the supernatant cultures previously filtered through 0.22 µm by High Performance Liquid Chromatography (HPLC), using a mobile phase of 5 mM sulfuric acid in water at 0.6 mL/min, and the stationary phase of organic acid Column Aminex^®^ HPX-87H Ion Exclusion Column at 50 °C [42]. For H_2_ quantification, the pressure generated (P) was measured in the headspace vial (V) using the manometer Omega HHP350 and subsequently converted into gas volume (V’) at atmospheric pressure (P’). Relative H_2_ concentration was analyzed by gas chromatography (GC) in a gas chromatographer equipped with a Poraplot Q Plot FS 25 × 53 column and a thermal conductivity detector (TCD) (Bruker 450 Daltonik GmbH, Bremen, Germany). The injector and detector were maintained at 250 and 150 °C respectively and the Ar carrier gas flow rate was maintained at 10 mL min^− 1^. The optical density (OD) was measured with a Thermo Corporation Genesys UV spectrophotometer at 570 nm of wavelength and it was used to estimate the CDW (1 OD_570_ = 0.33 g of cell dry weight [CDW]/L for *E. coli*). pH of the filtered media was measured with BASIC 20 + pHmeter (Crison Instruments).

## Results and discussion

### Enhancing L-malate production of the M4-ΔiclR/pck strain

**Fig. 1:**
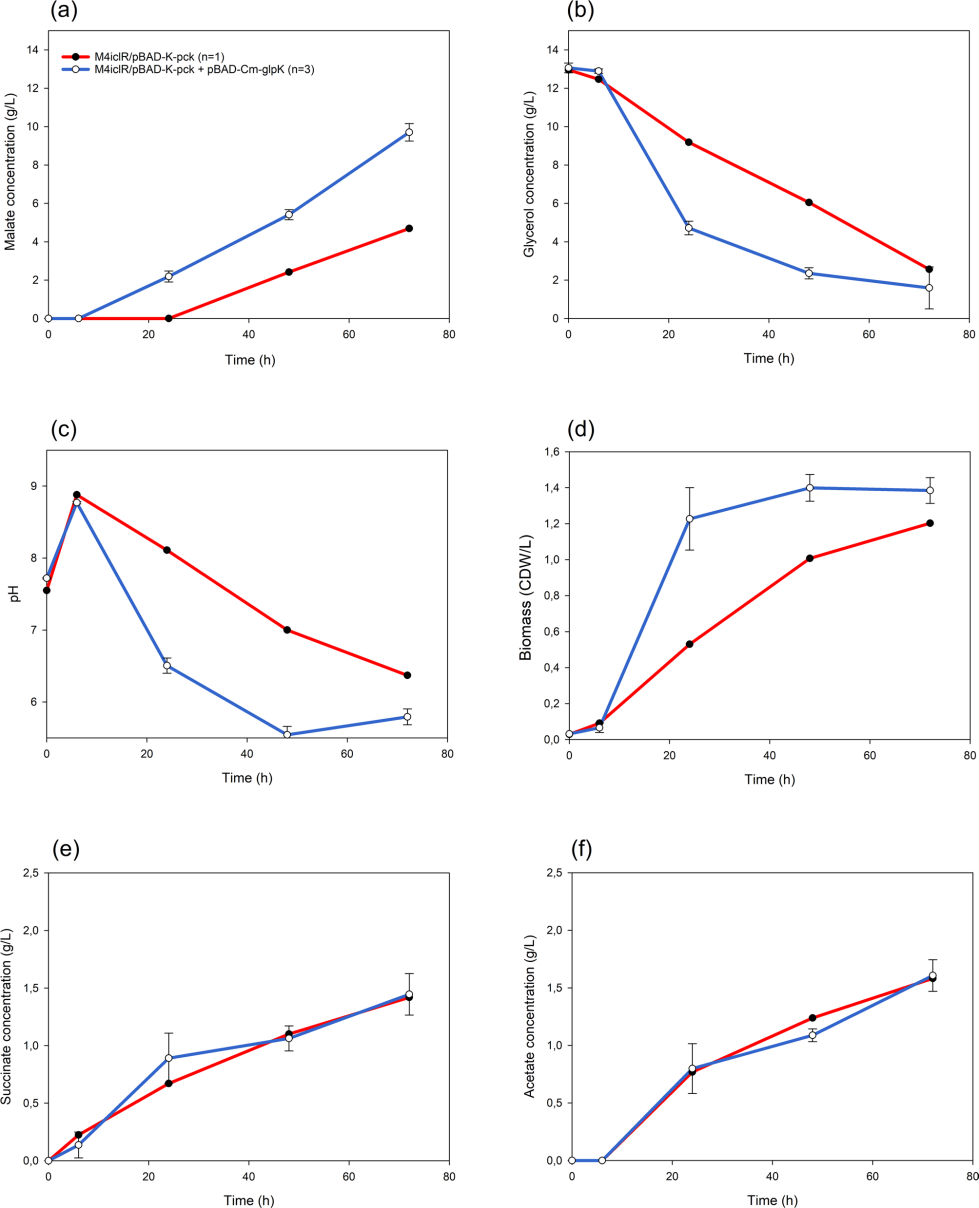
Comparative plots of the parameters analyzed during glycerol fermentation using the M4-∆iclR/*pck* control strain (*n* = 1), M4-∆iclR/*pck-glpK* (*n* = 3) at 0, 24, 48, and 72 h. Dot plots represent the average and standard deviation bars of: L-malate (a), glycerol (b), pH (c), biomass (d), succinate (e) in g/L, and acetate (f)

**Fig. 2:**
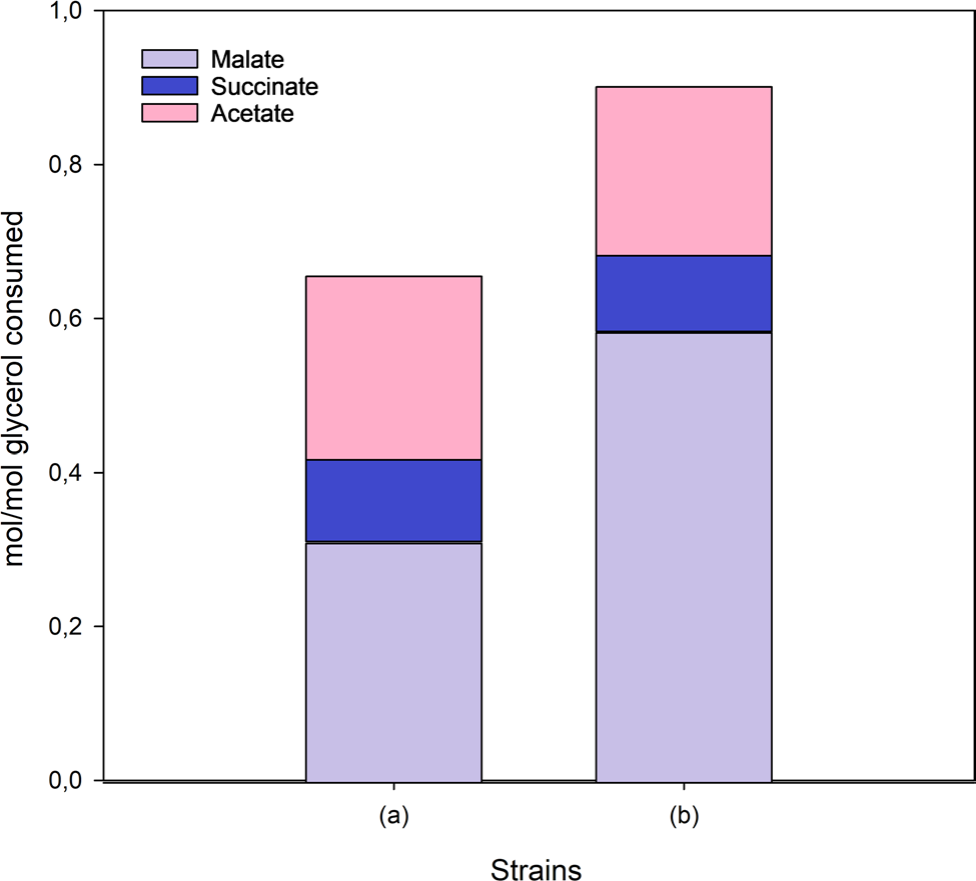
Cumulative bar graphs of C4 metabolites and acetate molar yields (mol produced/mol glycerol consumed) at 72 h by the M4-∆*iclR*/*pck* strain *n* = 1 (a) and M4-∆iclR/*pck-glpK* (*n* = 3) (b). Standard deviations for all parameters were < 0.09

Our initial objective was to enhance L-malate production by utilizing metabolic engineering on the M4-*ΔiclR/pck* strain [27]. We hypothesized that glycerol uptake could be a bottleneck in the process. Glycerol enters the cell via facilitated diffusion through the glycerol transporter protein (GlpF), and is then assimilated into cellular metabolism through phosphorylation by the glycerol kinase enzyme (GlpK) [49]. To test our hypothesis, we tried to enhance glycerol uptake by overexpressing the *E. coli glpK* (Table S1) in the previously reported genetic background (M4-∆*iclR*/*pck*) and using pure glycerol in Erlenmeyer Flasks (Fig. 1). We found that L-malate production and molar yields significantly improved in the strain overexpressing the *glpK* gene (M4-∆*iclR*/*pck*-*glpk* strain), reaching 9.71 ± 0.46 g/L at 72 h, while the control M4-∆*iclR*/*pck* strain produced 4.69 g/L of L-malate, a value similar to that reporter by [27], and consequently half of the production of the strain that additionally overexpressed *glpk* (Fig. 1a). This improved performance is indeed related to the predicted rapid uptake and consumption of glycerol, as evidenced by the decrease in glycerol concentration in the culture, which fell from 13.06 ± 0.25 g/L in the inoculation medium to 4.72 ± 0.36 g/L after 24 h. This is almost half the amount observed in the reference strain (Fig. 1b). The pH decreased in line with the accumulation of L-malate in the culture medium, reaching 5.8 ± 0.1 in the M4-∆*iclR*/*pck*-*glpk* strain (Fig. 1c). The growth of the strain overexpressing GlpK was also higher at this point-time, but remained unchanged up to 72 h (Fig. 1d). Therefore, most of the glycerol consumed from 24 to 72 h was transformed into L-malate. Moreover, succinate (1.45 ± 0.18 g/L) and acetate (1.61 ± 0.12 g/L) were also produced from glycerol (Fig. 1e, f). The L-malate molar yield (mol/mol of consumed glycerol) after 72 h was also significantly higher (0.58 ± 0.05 mol/mol) than that of the control strain (0.31 mol/mol), and the total organic acids molar yield from consumed glycerol was 0.90 ± 0.05 (mol/mol) (Fig. 2).

The increase in biomass and L-malate production in the M4∆*iclR/pck-glpK* strain can be explained by its higher glycerol uptake rate compared with the reference strain, which does not overexpress the GlpK enzyme. In the M4∆*iclR/pck-glpK* strain, the increase in glycerol consumption rate during the log phase was 0.46 g. L-1.h⁻¹, which is significantly higher than that of the reference strain (0.21 g L⁻¹ h⁻¹). This higher carbon uptake led to increased biomass and L-malate production as an end product of the linearized TCA cycle. Although the final glycerol uptake is similar in both strains, the higher L-malate production at 72 h indicates an increased CO₂ intake in the stationary phase due to the anaplerotic reaction catalyzed by Pck (PEP + CO₂ → OAA + ATP).

### Optimizing L-malate production in the M4-∆iclR/pck-glpK strain

**Fig. 3:**
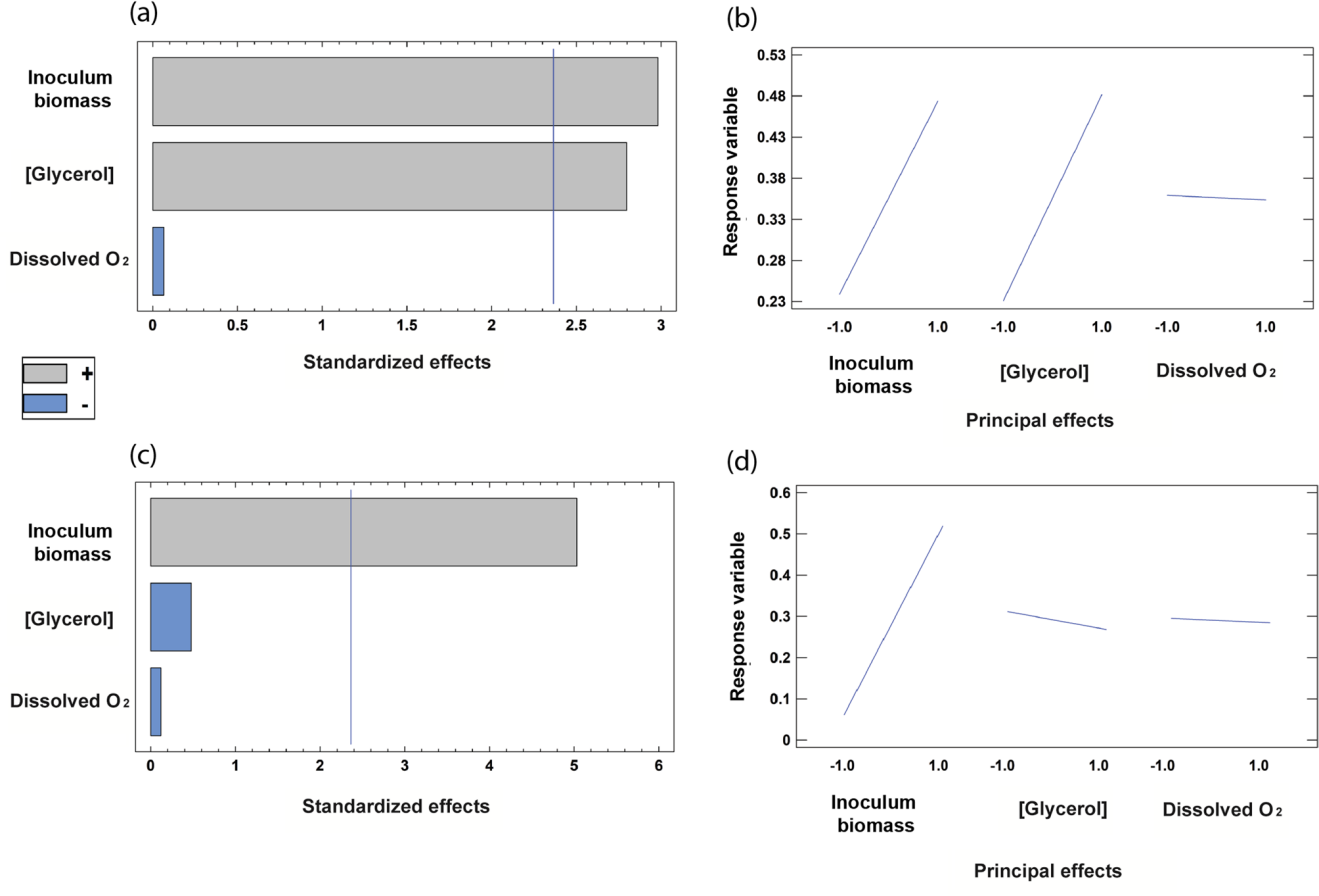
Pareto bar chart of the full-factorial screening for the identification of influential independent factors with L-malate yield as a response variable. The strain M4-∆*iclR*/*pck*-*glpK* was assayed using crude (a, b) or pure (c, d) glycerol. Initial glycerol concentration, biomass, and % dissolved oxygen were the response variables. Standardized (a, c) and principal (b, d) effects are shown. The vertical blue line represents the statistically significant value with a 95% confidence interval. The bars of components with a negative effect on malate yield are in blue, and those of components that have a positive effect on malate yield are shown in grey

The optimization of culture conditions is critical to scale up a biotransformation process from Erlenmeyer Flasks to bioreactors. To this end, we used the Micromatrix microbioreactor platform (Applikon, Netherlands), which allows the simultaneous monitoring and control of temperature, oxygen, and pH using small working volumes (3 mL in our case). A full-factorial screening was designed for the identification of independent factors that significantly influenced the L-malate molar yield at 24 h as a response variable using the optimized strain M4-*ΔiclR/pck-glpK* (Fig. 3). The factors evaluated were inoculum biomass, DO, and glycerol concentration in the initial culture medium (Table S2). This full factorial screening (Supporting information, Table S2) was carried out using either pure or crude glycerol to investigate possible differences in influential factors when using the two grades of glycerol. The analysis revealed that the initial biomass had a significant positive impact on L-malate yield in both cases (Fig. 3), and therefore OD_570_ 1.1 was selected as the inoculum biomass for further experimentation. Glycerol concentration was also a positive significant factor using crude glycerol within the analyzed concentration range (9 to 15 g/L) (Fig. 3a, b), but no statistical differences were found with pure glycerol (Fig. 3c, d). This effect is likely due to components other than glycerol in the crude glycerol extract. On the other hand, controlled DO at a range from 20% to 100% was not a significant factor using either crude or pure glycerol, and therefore, 20% DO was chosen for scaling up the process (Fig. 3). The inoculum biomass and crude glycerol concentration were also significant factors when pH and final biomass were set as response variables (data not shown).

### Dark fermentation: scaling up the production of L-malate in a bioreactor and in silico metabolic modeling and flux balance analysis (FBA)

To scale up L-malate production, the mini-bioreactor system MiniBio (Applikon^®^ Biotechnology) was used for monitoring and/or control of pH, temperature, and dissolved oxygen. Based on the results of the full-factorial screening previously described, the following parameters were set: DO controlled at 20% of air saturation, and an inoculum of induced bacteria at an OD_570_ of 1.1 (0.363 g CDW/L). The minimal medium was first supplemented with ∼12.5 g/L pure or crude glycerol as the only organic carbon source to test the scalability from Erlenmeyer flasks [27].

**Fig. 4:**
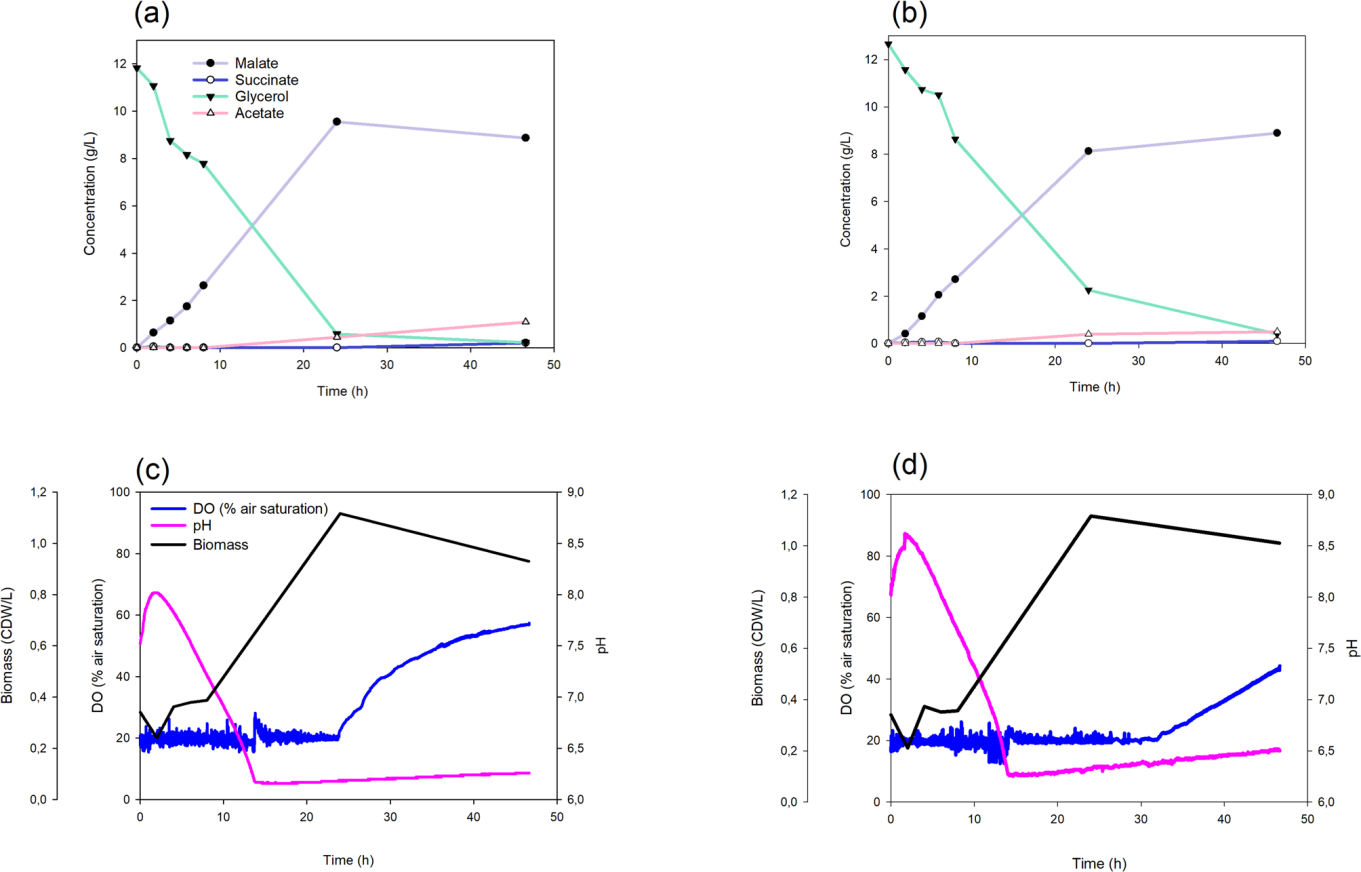
Comparative fermentation of pure and crude glycerol by the strain M4-∆*iclR*/*pck*-*glpK* strain in the Minibio bioreactor system (*n* = 1). Scatter plots showing the concentration of metabolites using crude (a) or pure (b) glycerol. Scatter plots of DO, pH, and biomass using crude (c) or pure (d) glycerol as a carbon source

These assays showed that L-malate reached similar maximal concentrations in the bioreactor as in the Erlenmeyer flasks (9.55 g/L with crude glycerol and 8.89 g/L with pure glycerol), but with higher productivity, obtaining the maximum values at 24 and 48 h, respectively, versus 72 h in Erlenmeyer flasks (Fig. 4a, b). Almost all of the crude glycerol had been consumed by 24 h, concomitantly with the maximum production of L-malate (Fig. 4a). As previously reported by [27], once the glycerol had been consumed, the bacteria used up the L-malate and decreased in the culture medium. In contrast, pure glycerol was fully consumed by 48 h, coinciding with the maximum L-malate production. (Fig. 4b). This suggests that the composition of the crude glycerol extract is suitable not only for *E. coli* growth [42], but also for L-malate production. Regarding pH, similar trends were observed when using either type of glycerol. There was an increase over three hours concomitant with a decrease in biomass. This was followed by acidification alongside malate production (Fig. 4c, d). In these experiments, pH was monitored but not controlled. The main reason for this was to keep dark fermentation as simple as possible. Given that the L-malate yield was quite high (0.65 ± 0.13 mol/mol) when 13 g/L of crude glycerol was used, we reasoned that scaling up would be easier and cheaper without pH control.

Oxygen was consumed steadily at the set point until the carbon source was depleted. At this stage, oxygen consumption ceased, and its concentration in the culture medium increased (Fig. 4c, d). These results support the use of the engineered *E. coli* strain to consume crude glycerol from a biodiesel refinery and produce L-malate. This process is even more efficient than using pure glycerol.

In order to investigate the metabolic rewiring of the strains used in this work, the experimental data were applied to metabolic reconstructions and simulations. For this, an iterative constraint matrix approach was performed to evaluate extracellular compounds by calculating metabolic fluxes using a metabolic-based model to generate the final metabolic flux distribution inside the cell under different scenarios. This allowed us to predict some non-experimentally measured parameters [50]. The strategies of metabolic engineering, together with the optimization of the culture conditions (glycerol grade, reactor configuration, and DO), revealed that fluxes from glycerol assimilation towards L-malate production have been improved from *E. coli* M4-*ΔiclR* (with pure glycerol medium) to the final *M4-ΔiclR/pck-glpK* mutant (with crude glycerol medium) (Table 1). FBA calculations reveal that CO_2_ production (Ex_ CO_2_) lowers in each step of optimization, and actually, becomes a net uptake of 3.13 mmol/g CDW h in M4-*ΔiclR/pck-glpK* strain. This in silico model indicates that there must be a net carbon uptake in the optimized strain because it is necessary for the carbon closure value (1.05) (Table 1). In theory, this additional uptake could come from CO_2_ or HCO_3_^−^ from crude glycerol, which were not experimentally measured. Regardless, the optimized metabolic phenotype displays minimized biomass, and maximized L-malate production and glycerol consumption. These differences are also apparent by examining the energetic metabolic pathways fluxes (mmol/g CDW in bracket): in the M4*-ΔiclR/pck-glpK* strain with crude glycerol, the source of ATP synthesis increased in glycolysis by phosphoglycerate kinase (PGK) (17.4), PCK (PPCK in Escher-map and CobraPy model) (13.2) when compared to the M4-*ΔiclR* strain with PGK (13.6) and PPCK (1.27). In contrast, the ATP Synthase (ATPS4r) activity diminished from 83.5 to 47.88 in the optimized strain (Table 1), which indicates that the oxidative phosphorylation is lower in this case. Flux variability analysis for ATP-related reactions shows that the flux range for PPCK in M4-*ΔiclR* was very low (1.27–3.48) and 0 in all of the strains overexpressing PPCK (Metabolic model results are available in Data availability statement). These results indicate that there is a unique pFBA solution for ATP synthesis in all of the constraints based-models for the strains overexpressing PPCK. In this strain, Pck is predicted to transform phosphoenolpyruvate (PEP) into oxalacetate (OAA), contributing to CO_2_ assimilation and ATP production. This is consistent with previous reports that *E. coli* overexpressing Pck with glucose in anaerobiosis had an intracellular ATP concentration that was approximately twice as high, increasing the ATP/ADP ratio via substrate phosphorylation rather than oxidative pathways, and this occurred concomitantly with CO₂ fixation [51, 52]. Our in silico analysis corroborates these results when using the optimized strain with crude glycerol in anaerobiosis, and also explains why a high O₂ concentration is not a significant factor, as was experimentally deduced from the full factorial design of experiment (Fig. 3). Regarding NADH production, GAPD fluxes increased from 13.8 to 17.4 and diminished in the NADH transhydrogenase (NADHTRHD) from 51.9 to 30.3 in the M4-*iclR* and M4-*iclR*/*pck-glpK* strains respectively (Table 1), confirming the NADH production is balanced with the malate dehydrogenase (MDH) activity.

**Table 1:** Metabolic flux (mmol/g CDW h) calculated by flux balance analysis (FBA) in the *E. coli* engineered strains, using pure or crude glycerol and in 50 mL-Flask or 150 mL-Bioreactor of metabolites and gases (O_2_ or CO_2_), ATP and NADH reactions

Reactions	Glycerol grade	Pure				Crude
	Strains	M4-iclR	M4-iclR/pck	M4-iclR/pck-glpK		
	Scale	50 mL -Flask	50 mL -Flask	50 mL -Flask	150 mL Biorreactor	
**Metabolites (C2-C4) and Biomass**	Glycerol	-20.0	-20.0	-20.0	-20.0	-20.0
	Malate	-0.11	5.45	5.78	10.10	12.79
	Biomass	0.48	0.26	0.25	0.16	0.15
	Acetate	1.49	6.38	3.26	1.76	2.29
	Succinate	3.10	2.14	1.39	0.01	0.21
**Oxygen and Carbon dioxide**	O_2_	-35.50	-21.96	-30.56	-29.37	-19.58
	Ex_CO_2_	24.59	5.75	14.37	9.45	-3.13
	Carbon Closure	0.61	0.90	0.76	0.84	1.05
**Atp_c producing**	ATPS4r	83.51	51.8	73.91	72.5	47.88
	PGK	13.61	19.3	14.89	13.83	17.43
	PPCK	1.268	6.28	6.48	10.54	13.23
	PYK	11.38	12.49	7.912	2.973	3.89
**NADH producing**	ALDD2x	1.765	6.38	3.26	1.77	2.29
	BIOMASS_Ecoli_core_w_GAM	0.55	0.92	0.87	0.55	0.55
	GAPD	13.83	19.3	14.89	13.83	17.43
	NADTRHD	51.92	15.51	42.54	51.92	30.3
	PDH	2.533	11.75	7.22	2.53	3.46

The fluxes values are represented in Figure S2. Negative values refer to reaction direction of uptake/consuming or opposite direction of physiological the reaction. Carbon closure refers to carbon balance, the value 1.0 refers to 100% carbon balance explained in the model

Reactions:

ATPS4r adp_c + 4.0 h_e + pi_c <=>atp_c + h2o_c + 3.0 h_c

PGK 3pg_c + atp_c <=>13dpg_c + adp_c

PYK adp_c + h_c + pep_c atp_c + pyr_c

GAPD g3p_c + nad_c + pi_c <=>13dpg_c + h_c + nadh_c

MDH mal__L_c + nad_c h_c + nadh_c + oaa_c

ME1 mal__L_c + nad_c co2_c + nadh_c + pyr_c

NADTRHD nad_c + nadph_c nadh_c + nadp_c

PDH coa_c + nad_c + pyr_c accoa_c + co2_c + nadh_c

ACALD acald_c + coa_c + nad_c <=>accoa_c + h_c + nadh_c

NADH16 4.0 h_c + nadh_c + q8_c 3.0 h_e + nad_c + q8h2_c

In summary, FBA analysis provides insight into metabolism, confirming that the engineered strain is a crucial step in optimizing crude glycerol fermentation. The model predicts a minimal O_2_ consumption by reducing metabolic energetic pathways, increasing the efficiency of C and energy utilization (avoiding fermentative end-products), and maximizing the C-flux towards L-malate. This optimized strain facilitates the scaling-up and the application of different bioprocess operations to increase L-malate production and productivity.

### Effect of crude glycerol concentration on the production of L-malate in bioreactor and fed-batch fermentation

**Fig. 5:**
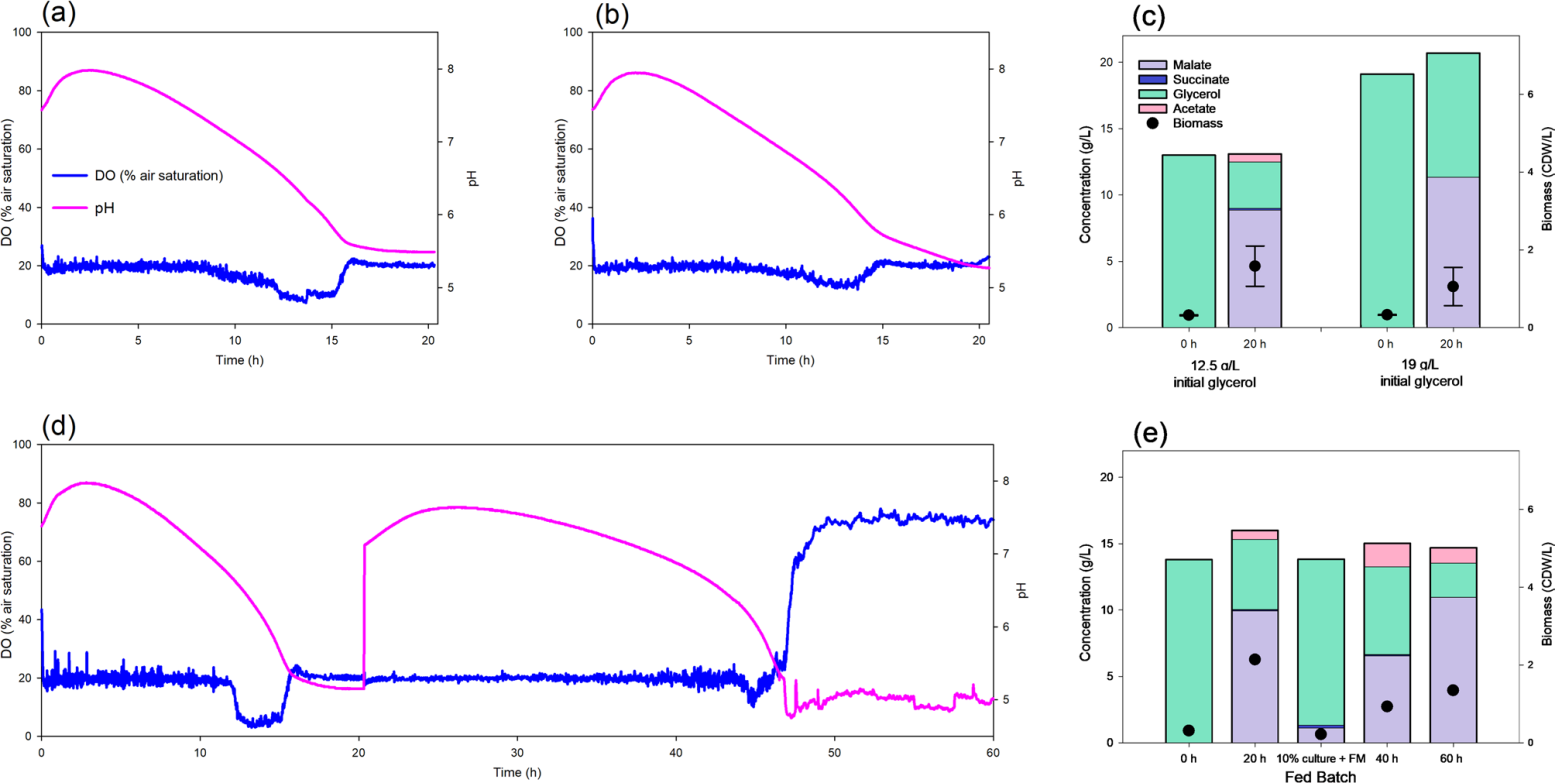
Fermentation of crude glycerol by the M4*ΔiclR/pck-glpK* strain. Dissolved oxygen and pH evolution using a culture medium supplemented with glycerol 12.5 g/L (a) or 19 g/L (b). (c) Bar diagram showing the concentrations of metabolites and biomass at 0 and 20 h (*n* = 3). (d) Dissolved oxygen and pH in a fed-batch fermentation (e) Bar diagram of the concentration of metabolites and biomass in the fed-batch fermentation. At 20 h 90% of the fermented culture was removed and refreshed with the same volume of the initial medium for a new batch

We continued to explore the optimization of the biotransformation in bioreactor comparing two different crude glycerol concentrations (12.91 ± 1.71 g/L and 19.12 ± 0.15 g/L) to set the optimum (Fig. 5). We found that L-malate production and yield at 24 h was higher using 19 g/L of glycerol (11.41 ± 2.88 g/L and 0.80 ± 0.09 molar yield), with no succinate or acetate production. To the best of our knowledge, this is the highest *E. coli* L-malate production using either pure or crude glycerol. However, almost half of the initial glycerol remained in the culture medium (Fig. 5c). On the other hand, when 12.9 g/L of glycerol was used, L-malate concentration and molar yield were slightly lower (8.94 ± 1.54 g/L and 0.65 ± 0.13 mol yield respectively), but 75% of glycerol was consumed (Fig. 5c). The pH evolution and oxygen intake were similar when using either concentration (Fig. 5a and b), with the highest L-malate concentration correlating with the lowest pH. Therefore, a better yield or L-malate concentration will depend on the initial glycerol concentration. Depending on the downstream application of the L-malate produced in the fermentation, either initial glycerol concentrations would be appropriate, since a lower concentration leads to a higher L-malate yield, and a higher concentration leads to a higher L-malate concentration.

At an industrial scale, fed-batch fermentation is a usual strategy [43]. To test the applicability of this strategy, one fed-batch fermentation was also carried starting with 12.5 g/L of glycerol and removing 90% of the culture medium after 20 h the inoculation and adding the same volume of fresh M9 crude glycerol medium (Fig. 5d and e). In this assay, the pH increased slightly to 7.8, and then dropped to 5 between 24 and 48 h, showing a similar trend to that observed between 0 and 24 h. Oxygen consumption remained constant up to 48 h, after which it decreased (Fig. 5d). In the first step, production of L-malate was also similar to that previously obtained in the single batch fermentation, reaching 10.07 g/L (Fig. 4d). However, L-malate concentration after 20 h of the second batch was significantly lower (6.68 g/L) but increased up to 11.09 g/L at 40 h. The production rate was lower, although the final concentration was higher in this second batch. This difference in production rate was probably due to the initial biomass in the first (OD_570_ = 1.1) and second (OD_570_ = 0.6) batches. Further studies would be needed to adjust the optimal biomass concentration by, for instance, removing less than 90% of the culture medium in the second batch. This study therefore validates the scaling up of L-malate production in bioreactors using *E. coli* and crude glycerol.

### Bio-H_2_ production by photofermentation using the L-malate-enriched culture medium from glycerol dark fermentation

**Fig. 6:**
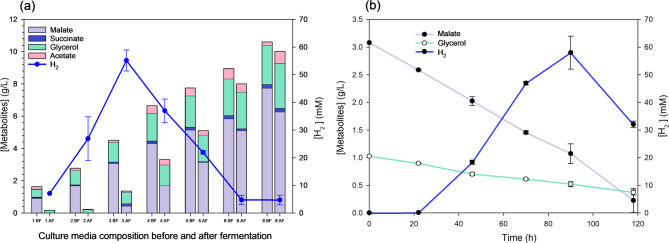
H_2_ production by photofermentation with *R. capsulatus* using L-malate from dark fermentation with *E. coli* (a) Stacked bar chart of metabolites concentrations (Y left axis) before (BF) and after (AF) photofermentation at 96 h using RCV L-malate-medium supplemented with 1, 2, 3, 4, 5, 6 and 8 g/L of L-malate from *E. coli* fermentation (X axis). H_2_ produced after fermentation is also shown (Y right axis). (b) Dot and line plots of malate and glycerol concentrations (Y left axis) and H_2_ production (Y right axis) at 22, 48, 72, 96, and 120 h after inoculation (0 h)

One of the drawbacks of biotechnological L-malate production is the purification and downstream processes required to obtain the highly purified extracts needed for certain industrial applications. Nevertheless, the fermented medium obtained from crude glycerol by DF in this study contains mainly L-malate, as well as small amounts of succinate, acetate, and non-consumed glycerol. This medium can be used in an additional fermentative process to produce an added-value product, such as bio-H_2_. This would be of particular interest in a biodiesel refinery, as it would transform a by-product into another clean energy source, creating an integrated process capable of producing two clean bioenergy sources from biomass: biodiesel and bio-H_2_. In this study, we propose a procedure that uses non-purified, crude glycerol to produce an L-malate-enriched medium. This medium is then used to formulate an RCV culture medium for producing bio-H₂ via photofermentation using the *R. capsulatus* S2 strain. To this end, we assayed increasing final concentrations of L-malate from 1 to 8 g/L in the RCV L-malate medium. We found that *R. capsulatus* grew and produced H₂ in all the conditions except when 6–8 g/L of L-malate was used, where H₂ production was negligible (< 5 mmol/L). The optimum was achieved using 3 g/L of L-malate with a H_2_ production of 55.14 ± 3.80 mmol/L and 62.97 ± 5.85 mmol H_2_/g CDW) (Fig. 6a). The analysis of the culture after photofermentation showed that the consumption of organic acids was almost 90% with 1 and 2 g/L L-malate in RCV medium, and 73% was consumed with 3 g/L L-malate. However, when higher L-malate concentrations were used, very low bio-H_2_ synthesis and growth were observed, probably due to an inhibition by the excess of L-malate or other components of the spent M9 culture medium. In this sense, although there was some L-malate consumption, most of the glycerol remained after photofermentation (Fig. 6a). In the 3 g/L L-malate - RCV medium, not only L-malate, but also acetate, succinate, and most of the glycerol content were consumed, which represents 87% of the carbon sources (Table 2). This means that the yield was around 1.5 mol H_2_/mol (C2-C4 consumed) and the productivities 0.6 mmol/L h and 0.7 mmol/g CDW h. To find out the optimum fermentation time point, the culture medium containing 3 g/L of L-malate was tested every 24 h from inoculation to 120 h. This analysis showed that 92 h was the optimal time point, at which 58.0 ± 6.0 mmol/L of bio-H₂ was achieved. Bio-H₂ dropped from 92 to 120 h (Fig. 6b), probably due to some remaining hydrogenase activity in the *R. capsulatus* strain used in this study. For example, it was reported that *R. capsulatus* B10 wild type uses 35 mM lactate and 7 mM sodium glutamate in a 1 L photo-bioreactor yielded 1.45 mol H_2_/mol lactate [53], which is very similar to the yield obtained in this work (1.48 mol H_2_/mol with a consumption of 36.5 mM C2-C4 compounds in this work) (Table 2). In terms of productivity [54], reported 0.56 mmol H_2_​/L h using *R. capsulatus* DSM 1710 at 27.5 °C with light power 287 W/m^2^, which are similar conditions to those of this work (0.59 mmol H_2_​/L h). Other *R. capsulatus* strain (KU002) produced 1264 mL H_2_​/L, a similar value to that obtained in this work (1041 mL/L) but with higher productivity (XXX6.8 mL H_2_​/L h). In this case, the author also used L-malate as a carbon source and improved the productivity by optimizing pH, temperature, and light intensity [53]. Recent publications have used several carbon sources (glucose, acetate, butyrate), obtaining variable productivity values (0.15–2.3 mmol/L h) [55, 56] reported a higher productivity (1.388 mmol H_2_/L h OD and 1500 mL/L culture) using the S2 strain and similar conditions to those of this work (RCV with 30 mM L-malate in diazotrophic conditions, 30 °C at 63 h). This difference is probably due to the higher light intensity (60 W) used by Barahona et al.

**Table 2:** Concentrations and standard deviations of the data shown in Fig. 6b

	0 h	92 h	
*C2*,* C3 and C4 source*	mM	mM	*Consumed* *C2*,* C3 and C4* *mM (%)*
L-Malate	24.02 ± 0.25	2.22 ± 1.55	21.80 (90.8%)
Succinate	0.84 ± 0.7	ND	0.84 (100%)
Acetate	3.29 ± 1.84	ND	3.29 (100%)
Glycerol	13.70 ± 0.79	3.17 ± 1.4	10.53 (76.9%)
Total	41.9	5.4	36.47 (87.1%)

Another widely used PNSB in hydrogen production is the *R. sphaeroides* species. This bacterium has been reported to produce hydrogen at a broad range of rates (0.29–8.7 mmol/L h) using different organic compounds (acetic, butyric, xylose, glucose, lactate, L-malate), or food wastes (oil palm, brewery effluent, restaurant effluent, etc.) [35]. The wide range of substrates, bioreactor configurations, and environmental conditions (including light intensity, temperature, pH, etc.) significantly affects H_2_ production and productivity, as also described for *R. capsulatus* and other PNSBs [55, 57]. Nevertheless, to the best of our knowledge, no publications have reported the consumption of glycerol and L-malate for H₂ production in *R. capsulatus* or other PNSBs. These evidences make this work very interesting in terms of productivity, reproducibility, and the scalability of photofermentative H_2_ process when a defined L-malate medium is used. The identification and construction of new PNSB strains by genetic engineering [58], together with bioprocess engineering studies, are promising strategies to improve H_2_ rates [41, 56, 58].

## Conclusion

In this work, we propose a two-step integrated process of DF and PF to produce bio-H_2_ from crude glycerol. We engineered an *E. coli* strain capable of producing up to 11.5 g/L of L-malate by DF. We also found that the optimal proportion of DF medium for PF is a volume containing 3 g/L of L-malate, which means that 1 L of DF medium will lead to more than 3 L PF culture medium. In this approach, L-malate purification is not only unnecessary but also inconvenient, since *R. capsulatus* uses also the non-consumed glycerol as well as the small amounts of succinate and acetate from dark fermentation. This two-step integrated system for glycerol ◊ L-malate ◊ bio-H_2_ production has many advantages in terms of reproducibility and scalability. To improve bio-H_2_ production, the RCV-L-malate medium produced by DF would be tested in other wild-type or genetically enhanced PNSB strains.

## Supplementary Information

Below is the link to the electronic supplementary material.

Supplementary Material 1. Table S1. Strains, plasmids, and oligonucleotides. Table S2. Parameters and conditions assayed in the full-factorial screening for optimization of L-malate production. Figure S1. L-Malate yield (mol L-malate/mol consumed glycerol) obtained in the full-factorial screening for optimization of L-malate production. Figure S2. Full visualization of the reactions in the Escher-map obtained from metabolic flux models.

## Data Availability

Metabolic models in Python codes are available in the GITHUB open repository in the following link: https://github.com/Valle-82/FBA\_E.coli\_M4\_iclR.git.

## Abbreviations

Bio-H2: Bio-hydrogen
DF: Dark fermentation
DoE: Design of experiment
FBA: Flux balance analysis
HVO: Hydrotreated vegetable oil
OAA: Oxalacetate
PEP: Phosphoenolpyruvate
PF: Photofermentation
PGK: Phosphoglycerate kinase
PNSB: Purple non-sulfur bacteria
PPCK: Phosphoenol pyruvate carboxykinase
SCFAs: Short chain fatty acids
TCA: Tricarboxylic acid cycle
VFAs: Volatile fatty acids
