# Supplementary material for: Production of hydrogen from crude glycerol via an integrated process of L-malate biosynthesis by Escherichia coli and photofermentation by Rhodobacter capsulatus
Source: Microb Cell Fact. 2025 Nov 10;24:230. doi: 10.1186/s12934-025-02866-y (PMC12599024; doi:10.1186/s12934-025-02866-y)

**Supplementary material**

**Table S1: Strains, plasmids, and oligonucleotides**

| Strains | Genotype | Source/ reference |
| --- | --- | --- |
| *E. coli* M4-∆*iclR*/*pck* | BW25113∆*sdhA*::FRT ∆*ack-pta*::FRT ∆*pox*::FRT ∆*iclR*::FRT harbouring the pBAD-Kan-*pck* cloning vector | This study |
| *E. coli* M4-∆*iclR*/*pck*-*glpK* | BW25113∆*sdhA*::FRT ∆*ack-pta*::FRT ∆*pox*::FRT ∆*iclR*::FRT harbouring the pBAD-Kan-*pck* and pBAD-Cm-*glpK* cloning vectors | This study |
| *Rhodobacter capsulatus S2* | S2 Δ*hupAB*, P_hupA_:::lacZ (Rif ^R^ Kan ^R^) | (Barahona *et al.*, 2016) |
| Plasmids | **Features** |  |
| pBAD-Kan-*pck* | pBAD-18-kan vector with the phosphoenol pyruvate carboxykinase ORF (*pck*) from *E. coli* cloned in the *Nhe*I (5’) and *Eco*RI (3’) sites. Kanamycin resistance. | (Soto-Varela *et al.*, 2021) |
| pBAD-Cm-*glpK* | pBAD-18-Cm vector with the phosphoenol pyruvate carboxykinase ORF (*pck*) from *E. coli Kpn*I (5’) and *Hind*III (3’) sites. Chloramphenicol resistance. | This study |
| Oilgonucleotides | **Sequence** |  |
| pBAD-Fw | CTCTACTGTTTCTCCATACCCG |  |
| pBAD-Rv | TATCAGACCGCTTCTGCG |  |
| Kan Res | CGGCCACAGTCGATGAATCC |  |
| EcoRI-pck-Rv | ggGAATTCTTACAGTTTCGGACCAGCCGC | (Soto-Varela *et al.*, 2021) |
| pox Fw | GGCTGCTGTAAGACAAAAGTGG | (Soto-Varela *et al.*, 2021) |
| pox Rv | TCAAACAGATAGTTATGCGCGG | (Soto-Varela *et al.*, 2021) |
| iclR Fw | TTTGCTGCTCACACTTGCTC | (Soto-Varela *et al.*, 2021) |
| iclR Rv | GGTGTTCATTTGTCTGGGCTG | (Soto-Varela *et al.*, 2021) |
| glpK-Fw | GGGAAGCTTAGGAGGAATTAACCATGACTGAAAAAAAATATATCG | This study |
| glpK-Rv | GGGGGTACCTTATTCGTCGTGTTCTTCCC | This study |
| Glpk-kpnI-SD-Fw | GGGGGTACCAGGAGGAATTAACCATGACTGAAAAAAAATATACG | This study |
| Glpk-HindIII-Rv | GGGAAGCTTTTATTCGTCGTGTTCTTCCC | This study |

**Table S2.** Parameters and conditions assayed in the full-factorial screening for optimization of L-malate production using the Micromatrix microbioreactor platform. The strain used in this assay was M4-∆*iclR*/*pck-glpK* grown in the same culture medium (M9) except for the initial concentration of glycerol. The same assay was carried out either with pure or crude glycerol. The fermentation time was 24 h.

| Conditions (ID) | Initial biomass (OD_570 nm_) | Initial_[glycerol] (g/L) | Dissolved oxygen (%) |
| --- | --- | --- | --- |
| 1 | 0.60 | 12 | 60 |
| 2 | 0.60 | 12 | 60 |
| 3 | 0.60 | 12 | 60 |
| 4 | 0.10 | 9 | 20 |
| 5 | 1.10 | 9 | 20 |
| 6 | 0.10 | 15 | 20 |
| 7 | 1.10 | 15 | 20 |
| 8 | 0.10 | 9 | 100 |
| 9 | 1.10 | 9 | 100 |
| 10 | 0.10 | 15 | 100 |
| 11 | 1.10 | 15 | 100 |

**SUPPLEMENTARY FIGURES**

**
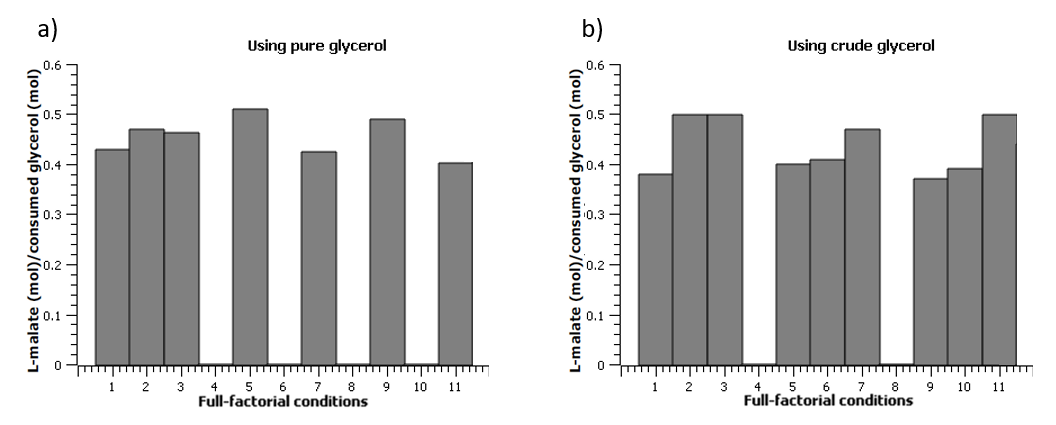
**

**Figure S1: L-**Malate yield (mol L-malate / mol consumed glycerol) obtained in the full-factorial screening for optimization of L-malate production. Results 1-11 were used as the response variable for full-factorial screening shown in Figure 3.

**Figure S2.** Full visualization of the reactions in the Escher-map obtained from metabolic flux models of the *E. coli* mutant strains: M4-∆*iclR* (a) and M4-∆*iclR*/*pck*-*glpK* (b). The colour scale refers to flux absolute values. Grey colour indicates no fluxes.


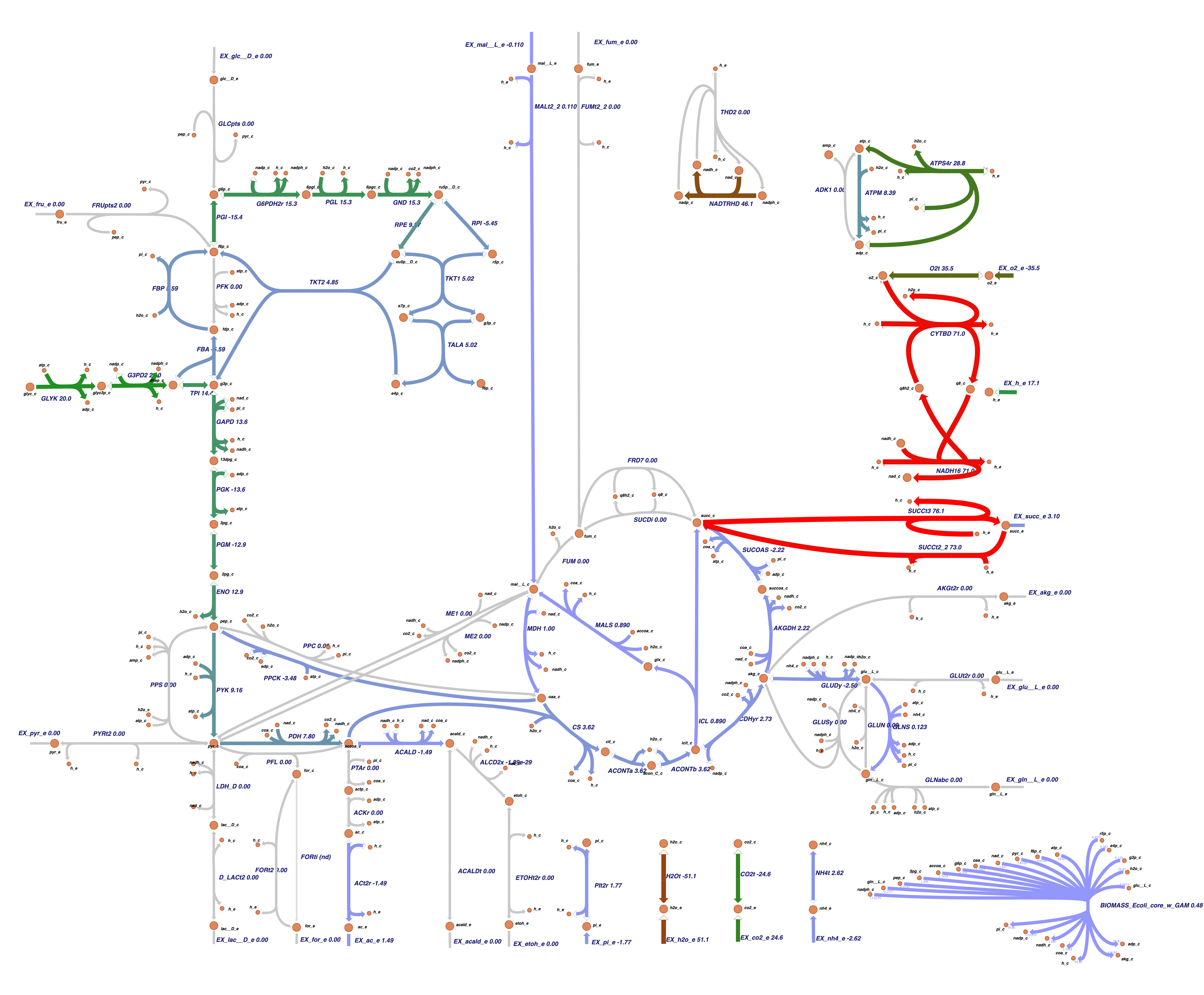

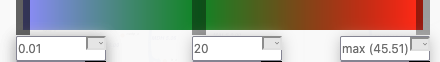


(a)

)

(b)


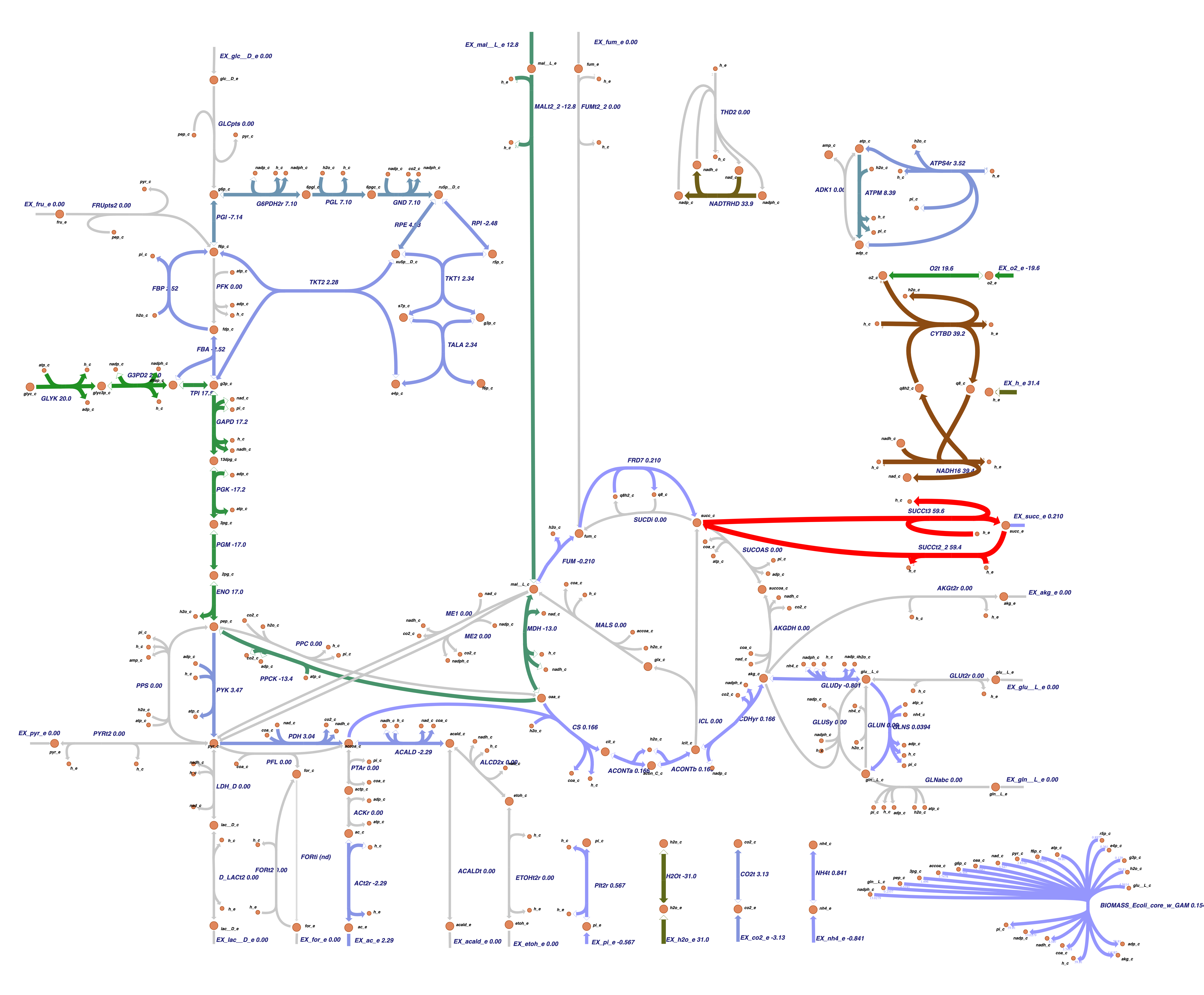

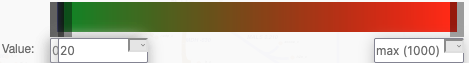

Supplement: Supplementary file 1 — Supplementary Material 1. Table S1. Strains, plasmids, and oligonucleotides. Table S2. Parameters and conditions assayed in the full-factorial screening for optimization of L-malate production. Figure S1. L-Malate yield (mol L-malate/mol consumed glycerol) obtained in the full-factorial screening for optimization of L-malate production. Figure S2. Full visualization of the reactions in the Escher-map obtained from metabolic flux models. [file 12934_2025_2866_MOESM1_ESM.docx]
